# Current Use and Discrepancies in the Adoption of Health-Related Internet of Things and Apps Among Working Women in Japan: Large-Scale, Internet-Based, Cross-Sectional Survey

**DOI:** 10.2196/51537

**Published:** 2024-07-31

**Authors:** Kirio Sasayama, Etsuko Nishimura, Noyuri Yamaji, Erika Ota, Hisateru Tachimori, Ataru Igarashi, Naoko Arata, Daisuke Yoneoka, Eiko Saito

**Affiliations:** 1 Sustainable Society Design Center Graduate School of Frontier Sciences The University of Tokyo Kashiwa Japan; 2 Faculty of Nursing Komazawa Women’s University Tokyo Japan; 3 Graduate School of Nursing Science St. Luke's International University Tokyo Japan; 4 Institute of Clinical Epidemiology Showa University Tokyo Japan; 5 Graduate School of Medicine The University of Tokyo Tokyo Japan; 6 Institute for Global Health Policy Research Bureau of International Health Cooperation National Center for Global Health and Medicine Tokyo Japan; 7 Department of Health Policy and Management Keio University School of Medicine Tokyo Japan; 8 Public Health, School of Medicine Medical Course Yokohama City University Yokohama Japan; 9 Center for Maternal-Fetal-Neonatal and Reproductive Medicine National Center for Child Health and Development Tokyo Japan; 10 Center for Surveillance, Immunization, and Epidemiologic Research National Institute of Infectious Diseases Tokyo Japan

**Keywords:** women's health, IoT, mHealth, mobile health, app, apps, application, applications, decision tree, internet survey, women, health, adoption, Internet of Things, survey, surveys, management, working women, worker, workers, employee, employees, occupational health, job, jobs, working, employed, usage

## Abstract

**Background:**

Demographic changes and a low birth rate have led to a workforce shortage in Japan. To address this issue, the government has promoted engagement of female employment. However, increased female employment can impact women's health. Using Internet of Things (IoT) and apps to manage women's health has gained attention, but few studies have focused on working women.

**Objective:**

This study aimed to clarify the current situation of working women and their use of IoT or apps to manage their health.

**Methods:**

A large-scale, nationwide internet survey was conducted among 10,000 female participants aged from 20 years to 64 years in Japan. Participants were recruited from a marketing research company’s active survey panel of 5.24 million members. The survey included questions about health status, sociodemographic factors, psychological characteristics, and the use of IoT or apps for health management. We compared perceived health status and reasons for current IoT use using t tests and assessed participant characteristics that predicted IoT use using the C5.0 decision tree algorithm. Ethical approval was granted by St. Luke's International University.

**Results:**

Among participants, 14.6% (1455/10,000) currently used IoT or apps, 7% (695/10,000) used them previously, and 78.5% (7850/10,000) had never used them. Current users (42.7 years old) were older than past users (39.7 years old). Discrepancies were observed between participants’ perceived health problems and the purpose for using IoT or apps, with 21.3% (2130/10,000) of all women reporting they experienced menstrual symptoms or disorders but only 3.5% (347/10,000) used IoT or apps to manage the same symptom. On the other hand, current users were more likely to use IoT or apps to manage nutrition-related problems such as underweight or obesity (405/1455, 27.8%). Device use was highest among current users, with 87.3% (1270/1455) using smartphones, 19.7% (287/1455) using smartwatches, and 13.3% (194/1455) using PCs. Decision tree analysis identified 6 clusters, the largest consisting of 81.6% (5323/6523) of non-IoT users who did not exercise regularly, while pregnant women were more likely to use IoT or apps.

**Conclusions:**

Our findings highlight the idea that woman with particular health problems (ie, menstrual symptoms or disorders and premenstrual syndrome) have lower use of IoT or apps, suggesting an unmet need for IoT and apps in specific areas.

## Introduction

As many other high-income countries have experienced, demographic changes in Japan have resulted in an aging population, and a low birth rate has led to a workforce shortage. To address this structural problem, in 2016, the Japanese government formulated a policy called “Promoting the Dynamic Engagement of All Citizens,” in which all citizens, regardless of gender, age, or disability, are encouraged to collectively play an active role in the workforce [1-3]. This policy included the promotion of female employment, and the employment rate of women aged 15 years to 64 years, which was 66.0% in 2016, had risen to 71.3% by 2021 [4]. Although the promotion of female employment has had a positive effect on the economy by increasing the working population [5,6], it has also brought changes in women's social and biological conditions.

For example, socially, it has induced changes in outcomes related to childbearing, such as lower fertility rates and advanced-age childbirth. In addition, long working hours and night shifts increase the biological risk of adverse pregnancy outcomes for pregnant women, including gestational hypertension, premature birth, and babies with low birth weight [7,8]. Furthermore, some women transitioning to menopause experience difficulties at work, such as poor concentration, fatigue, poor memory, depressed mood, melancholy, low self-confidence, and hot flashes, which affect some women’s work performance [9]. Although the severity of symptoms varies from person to person, these health problems have been strongly associated with decreased productivity [10,11]. For example, labor losses due to menstrual symptoms, which are specific to women, are estimated at ¥491.1 billion. Addressing women's health issues through health management and promoting the development of a social environment in which women can work comfortably is considered to lead to increased productivity and improved corporate performance [12].

Although past health initiatives have mainly focused on combating metabolic syndrome, it is believed that increasing initiatives for the well-being of women, who account for approximately 44% of all employees in Japan (2016), will lead to further revitalization of companies. Thus, women's health not only is an individual problem but also has the potential to affect society and the health of the next generation. Among efforts to tackle this issue, Internet of Things (IoT) and apps to manage and control women's health have attracted strong attention in the past decades [13-15].

The term IoT, invented by Kevin Ashton in 1999, is an interactive network of smart objects and devices used in our daily lives [16,17]. To date, various types of IoT devices have been developed, such as smartwatches, smart rings, smart clothing, and smart glasses, to name a few. There are also a wide variety of apps that promote behavioral change. For example, smartphone apps have been developed to improve postpartum weight in women with gestational diabetes mellitus [18,19], smart clothing has been designed to monitor body temperature and other health conditions for menopausal women [13], and smart wristbands monitor certain health indicators, such as physical activity, sleep, and heart rate of pregnant women [20,21]. These studies have shown the effectiveness of IoT in facilitating lifestyle behavior change and improving people’s health. IoT devices are comfortable, convenient, affordable, and effective. The customer base for wearable devices is expanding to include women with chronic conditions, carers, and older adults [22]. For example, hot flashes as well as their persistence and intensity experienced by menopausal women can be monitored at home and on the go, while sleep disturbances caused by menopause can be measured [13].

To date, however, few studies have analyzed “working” women’s health and their use of IoT.

This study aimed to identify the health problems of working women, the current use of IoT and apps for health management, and the characteristics of IoT and app users. To achieve this goal, we conducted a large-scale, nationwide, internet survey with women aged 20 years to 64 years from the general population in Japan.

## Methods

### Study Design and Participant Recruitment

A marketing research survey company was commissioned to recruit research participants from the largest active survey panel in Japan, consisting of Japanese women living in 47 prefectures across Japan. As of June 2022, the company that managed the panel had a total of 5.24 million active panel members (men and women) of various backgrounds [23]. All participants were aged between 20 years and 64 years and were able to respond to the survey questionnaire in Japanese. Working women in our study were defined as women who were working for payment under an employment contract at the time of the questionnaire, regardless of working style or working hours.

For this study, the sample size was set at approximately 10,000 women to allow disaggregated analyses by prefecture level. To determine the sample size by age group (20s, 30s, 40s, 50s, and 60-64 years), the well-being score among Japanese women from a World Value Survey (2021), in which 91.5% of women responded that they were “very happy” or “happy” [24], was used as the assumed proportion for all prefectures, assuming a type-1 error level of 0.05, statistical power of 80%, an acceptable margin of error of 1.3%, and a dropout or missing rate of 10%. To ensure national representativeness, a quota random sampling approach based on age at the time of the survey and prefecture population ratios obtained from the 2015 National Census were used. Participation was on a first-come-first-served basis, and the survey closed when the number of respondents reached the target sample sizes per age group and prefecture.

The survey was conducted from February 28, 2023, to March 7, 2023, and the target sample size was reached on the last day. Respondents were requested to answer every question to prevent missing values. Completed surveys were checked for duplicates based on the participants’ demographic information to avoid double responses. Data were received with personal information deleted. By the last date, 17,398 individuals had read the informed consent form on the survey website, and 16,219 had agreed to participate. The survey was completed when 10,000 surveys had been completed.

### Questionnaire

The questionnaire was developed based on a comprehensive literature review on well-being and other epidemiological surveys [25,26]. The survey items were divided into 3 modules: health; sociodemographic characteristics; and psychological characteristics, including well-being. We cited a questionnaire from a previous study that had investigated sociodemographic and health-related characteristics in a Japanese population [26]. The well-being questions in our study consisted of common inquiries widely used in various studies. Although not a single set of rating scale questionnaires has been specifically designed for the Japanese population, each item in our survey is nevertheless frequently used in numerous Japanese surveys [27]. The questionnaire also covered the use, frequency of use, purpose of use, and satisfaction level with IoT and apps to promote health. The first category focused on subjective well-being and included 6 questions. Participants were asked to rate their well-being on a scale of 0 to 10, where 0 indicated “not at all” and 10 indicated “completely.” The well-being questions used in this study were derived from the UK Office of National Statistics [28]. An important question in this questionnaire was whether the respondent used any health-related IoT device or app, which was the main interest of the study. The response options were categorized as current users, previous users, and never users. Those who indicated “current use” or “previous use” were further asked about the types of diseases or symptoms they were experiencing and the purpose of using IoT or apps to prevent or control those conditions. They were also asked about the frequency of use and the types of media they used, such as PCs, tablets, smartphones, and wearable devices. Additionally, participants who indicated “previous use” were asked about the reasons why they stopped using IoT or apps. All questions in the questionnaire were closed-ended with various formats, including single or multiple-answer formats, binary “yes/no” scales, nominal and ordinal scales, and Likert scale questions. Details of the study questionnaire are provided in Multimedia Appendix 1. There were 79 questions. Participants completed the survey via an app or email for each survey site with which they registered.

### Ethics Approval

Written informed consent was obtained from all participants following a full explanation of the study purpose, methods, and secondary use of the data. Participants were also notified that they would receive shopping points (¥40-¥100 [US $0.25-$0.63]) as a financial incentive and that the survey would be closed once the required sample size for all items had been answered. The privacy and confidentiality of participants’ information were strictly maintained throughout the study, in accordance with ethical standards and guidelines. Ethical approval as a study on human subjects was granted by the Ethics Committee of St Luke's International University (approval number 22-A089), and the study conformed to the principles of the Declaration of Helsinki.

### Statistical Analysis

Baseline data are reported as mean (SD) or proportion (%). The Kruskal-Wallis test was used to analyze continuous variables, and the Fisher exact test was used to analyze categorical variables. The Kruskal-Wallis test was chosen for its nonparametric nature, which makes it ideal for analyzing non-normally distributed continuous data. This makes it suitable for our data, which may not follow a normal distribution. The Fisher exact test is particularly preferred when the sample size is small, as it provides exact *P* values when the chi-square test’s assumptions are not met. These nonparametric tests ensure more accurate analysis in cases where parametric test assumptions are not applicable. To examine the discrepancy between the participants’ experiences with women-specific health problems and symptoms and the purpose of the current use of IoT and apps, the proportions with estimated 95% CIs were plotted as bar plots. We performed this same analysis by age categories, namely 20-29 years, 30-39 years, 40-49 years, and 50-64 years.

To characterize the participants and identify subpopulations (clusters) based on their background information, we used the C5.0 algorithm [29], which is a variant of the decision tree algorithm and a powerful and widely used tool for classification and prediction in a variety of fields, including business, health care, and social sciences. Compared with a logistic regression model for binary classification tasks, the C5.0 decision tree algorithm can present the results in a tree structure, making interactions between variables easily realized and facilitating visual inspection of the results. The C5.0 algorithm works by dividing the data into subsets based on the values of the input variables based on the data entropy then recursively dividing the data into smaller subsets until a final decision can be made about the classification of each data point. The algorithm can produce a decision tree based on the input variables and then branches out to create new nodes for each possible value of the variable. The smallest number of samples that must occur in at least 2 of the splits was set at 20, and winnowing for input variables was used after checking accuracy using 10-fold cross-validation iterations. The data set was randomly split into 80% training and 20% test data sets, and the prediction performance, including accuracy, sensitivity, specificity, positive prediction value, and negative prediction value, was checked on the test data set. Statistical analyses were conducted with R (version 4.2.1) software with the C50 package. For the statistical 2-sided tests, *P* values <.05 were considered statistically significant.

This survey was designed in accordance with the Checklist for Reporting Results of Internet E-Surveys (CHERRIES) [30].

## Results

### Survey Participants

The main characteristics of the study participants are presented in Table 1. A total of 10,000 working women were enrolled in Japan during the study period, from February 28, 2023, to March 7, 2023. Of the 10,000 participants (all working women), 1455 (14.6%) reported that they currently used IoT or apps, 695 (7%) reported that they had previously used IoT or apps, and 7850 (78.5%) reported that they had never used IoT or apps. The average age of the participants indicated that past users were the youngest (39.7 years old), followed by current users (42.7 years old) and never users as the oldest (43.7 years old). Among the 10,000 participants, 3600 (36%) were company employees (full-time employees). Among participants who currently used or had used IoT in the past, full-time employment was the most common; among those who had never used IoT, part-time employment was the most common (3041/7850, 38.7%).

**Table 1:** Basic characteristics of 10,000 Japanese working women recruited to complete the internet survey.

Characteristics			Total sample (n=10,000)		Experience with health-related IoT^a^ or app use							
					Current use (n=1455)		Past use (n=695)		Never used (n=7850)		*P* value	
Age (years), mean (SD)			43.25 (11.41)		42.69 (11.24)		39.70 (11.56)		43.66 (11.37)		<.001	
Satisfaction with current life (0-10), mean (SD)			6.61 (2.42)		6.84 (2.41)		5.99 (2.57)		6.63 (2.40)		<.001	
Level of perceived value of current life (0-10), mean (SD)			6.65 (2.35)		6.97 (2.41)		6.07 (2.52)		6.64 (2.32)		<.001	
Level of happiness felt yesterday (0-10), mean (SD)			6.78 (2.55)		7.04 (2.52)		6.24 (2.67)		6.77 (2.54)		<.001	
Level of anxiety felt yesterday (0-10), mean (SD)			5.17 (2.79)		5.27 (2.88)		5.31 (2.68)		5.14 (2.78)		.10	
Level of satisfaction with current workplace (0-10), mean (SD)			6.18 (2.51)		6.34 (2.59)		5.92 (2.51)		6.18 (2.49)		.001	
Level of satisfaction with leisure time (0-10), mean (SD)			6.65 (2.42)		6.86 (2.45)		6.13 (2.51)		6.66 (2.40)		<.001	
Currently pregnant, n (%)			337 (3.4)		70 (4.8)		50 (7.2)		217 (2.8)		<.001	
Marital status (living with spouse), n (%)			5546 (55.5)		829 (57)		394 (56.7)		4323 (55.1)		.32	
**Number of children living together, n (%)**												<.001
	None	5638 (56.4)		803 (55.2)		364 (52.4)		4471 (57)				
	1	2166 (21.7)		315 (21.6)		169 (24.3)		1682 (21.4)				
	2	1649 (16.5)		227 (15.6)		109 (15.7)		1313 (16.7)				
	3	457 (4.6)		89 (6.1)		40 (5.8)		328 (4.2)				
	4	67 (0.7)		14 (1)		8 (1.2)		45 (0.6)				
	≥5	23 (0.2)		7 (0.5)		5 (0.7)		11 (0.1)				
**Occupation, n (%)**												<.001
	Management or executive	67 (0.7)		17 (1.2)		7 (1)		43 (0.5)				
	Company employee (full-time)	3600 (36)		622 (42.7)		310 (44.6)		2668 (34)				
	Company employee	517 (5.2)		83 (5.7)		35 (5)		399 (5.1)				
	Company employee (temporary employee)	453 (4.5)		68 (4.7)		37 (5.3)		348 (4.4)				
	Public employee (excluding teaching staff)	267 (2.7)		60 (4.1)		26 (3.7)		181 (2.3)				
	Faculty member	220 (2.2)		35 (2.4)		24 (3.5)		161 (2.1)				
	Health care provider	614 (6.1)		78 (5.4)		42 (6)		494 (6.3)				
	Professional	39 (0.4)		9 (0.6)		7 (1)		23 (0.3)				
	Independent business/self -employed workforce	598 (6)		68 (4.7)		38 (5.5)		492 (6.3)				
	Part-time work	3625 (36.2)		415 (28.5)		169 (24.3)		3041 (38.7)				
**Working shift, n (%)**												<.001
	Permanent day shift	9245 (92.4)		1345 (92.4)		597 (85.9)		7303 (93)				
	Permanent night shift	212 (2.1)		44 (3)		46 (6.6)		122 (1.6)				
	Alternating shifts (both day shift and night shift)	543 (5.4)		66 (4.5)		52 (7.5)		425 (5.4)				
**Average work duration** **per day in the p** **revious month (hours), n (%)**												<.001
	<6	2908 (29.1)		342 (23.5)		156 (22.4)		2410 (30.7)				
	6-8	4496 (45)		692 (47.6)		316 (45.5)		3488 (44.4)				
	8-10	2235 (22.4)		364 (25)		191 (27.5)		1680 (21.4)				
	>10	361 (3.6)		57 (3.9)		32 (4.6)		272 (3.5)				
**Income (¥^b^), n (%)**												<.001
	<2 million	1103 (11)		117 (8)		67 (9.6)		919 (11.7)				
	2-4 million	2430 (24.3)		289 (19.9)		140 (20.1)		2001 (25.5)				
	4-6 million	2356 (23.6)		321 (22.1)		172 (24.7)		1863 (23.7)				
	6-8 million	1741 (17.4)		296 (20.3)		118 (17)		1327 (16.9)				
	8-10 million	1159 (11.6)		185 (12.7)		86 (12.4)		888 (11.3)				
	10-12 million	586 (5.9)		121 (8.3)		54 (7.8)		411 (5.2)				
	12-14 million	247 (2.5)		34 (2.3)		28 (4)		185 (2.4)				
	>14 million	378 (3.8)		92 (6.3)		30 (4.3)		256 (3.3)				
**Education, n (%)**												<.001
	Junior high school	150 (1.5)		11 (0.8)		13 (1.9)		126 (1.6)				
	High school	2708 (27.1)		296 (20.3)		132 (19)		2280 (29)				
	Junior college	1571 (15.7)		205 (14.1)		90 (12.9)		1276 (16.3)				
	Vocational school	1706 (17.1)		221 (15.2)		122 (17.6)		1363 (17.4)				
	Undergraduate	3574 (35.7)		646 (44.4)		310 (44.6)		2618 (33.4)				
	Graduate	291 (2.9)		76 (5.2)		28 (4)		187 (2.4)				
**Medical history, n (%)**												
	None	7826 (78.3)		1027 (70.6)		516 (74.2)		6283 (80)		<.001		
	Infertility^c^	194 (1.9)		56 (3.8)		26 (3.7)		112 (1.4)		<.001		
	Female cancer^d^	207 (2.1)		52 (3.6)		27 (3.9)		128 (1.6)		<.001		
	High blood pressure	507 (5.1)		104 (7.1)		45 (6.5)		358 (4.6)		<.001		
	Diabetes mellitus	195 (2)		44 (3)		27 (3.9)		124 (1.6)		<.001		
	Hyperlipidemia	327 (3.3)		64 (4.4)		26 (3.7)		237 (3)		.02		
	Stroke	47 (0.5)		17 (1.2)		11 (1.6)		19 (0.2)		<.001		
	Heart disease	57 (0.6)		12 (0.8)		6 (0.9)		39 (0.5)		.18		
	Chronic kidney disease	41 (0.4)		9 (0.6)		8 (1.2)		24 (0.3)		.002		
	Others	1007 (10.1)		184 (12.6)		54 (7.8)		769 (9.8)		<.001		
Smoking status, n (%)			1373 (13.7)		254 (17.5)		126 (18.1)		993 (12.6)		<.001	
Exercise twice a week^e^ (yes), n (%)			1832 (18.3)		487 (33.5)		155 (22.3)		1190 (15.2)		<.001	
**Alcohol intake, n (%)**												< .001
	Daily	1299 (13)		266 (18.3)		86 (12.4)		947 (12.1)				
	Occasionally	2998 (30)		498 (34.2)		295 (42.4)		2205 (28.1)				
	Never	5703 (57)		691 (47.5)		314 (45.2)		4698 (59.8)				
**Total average sleep duration per month (hours), n (%)**												.24
	<5	1178 (11.8)		165 (11.3)		88 (12.7)		925 (11.8)				
	5-6	3405 (34.1)		481 (33.1)		245 (35.3)		2679 (34.1)				
	6-7	3511 (35.1)		516 (35.5)		248 (35.7)		2747 (35)				
	7-8	1525 (15.2)		248 (17)		93 (13.4)		1184 (15.1)				
	>8	381 (3.8)		45 (3.1)		21 (3)		315 (4)				

^a^IoT: Internet of Things.

^b^A currency exchange rate of ¥1=US $0.006 is applicable.

^c^Including cases with unknown reasons for infertility.

^d^Cervical, uterine, ovarian, or breast cancer.

^e^Exercise for at least 30 minutes twice a week for at least 1 year.

Of the 10,000 participants, 3655 (36.5%) reported having experienced women-specific health issues or symptoms. In both the current and past IoT user groups, menstruation-related symptoms were the most common, reported by 401 (401/1455, 27.6%) and 177 (177/695, 25.5%), respectively. Among those who had never used IoT or apps, headache was the most common symptom (1928/7850, 24.6%).

In comparison with past and never users, current users expressed higher levels of satisfaction with their current lives, valued their current lives, and were satisfied with their leisure time. Furthermore, compared with the other groups, current users were more likely to have a household income of at least ¥6 million and were more likely to have completed graduate school within their group.

Pregnant women accounted for a small proportion of all women, including 4.8% (70/1455) of those currently using IoT or apps, 7.2% (50/695) of those who had used IoT or apps in the past, and 2.8% (217/7850) of women who had never used them.

### Assessment of Current Users, Previous Users, and Never Users

Table 2 shows the type and frequency of IoT and app use for current and past users. Regarding devices for IoT or apps for health purposes, the 3 most popular devices were smartphones (current users: 1270/1455, 87.3%; previous users: 535/695, 77%; *P*<.001), smartwatches (current users: 287/1455, 19.7%; 96/695, 13.8%; *P*=.001), and PCs (current users: 194/1455, 13.3%; previous users: 112/695, 16.1%; *P*=.10). Notably, there were significant differences in the frequency of use per week between current users and previous users (*P*<.001): The most common frequency was 6 days to 7 days for current users (716/1455, 49.2%), compared with only 2 days to 3 days for previous users (211/695, 30.4%).

**Table 2:** Type and frequency of use of health-related Internet of Things (IoT) and apps by current and past users among Japanese working women.

IoT or app use characteristics		Total sample (n=2150), n (%)	Experience with health-related IoT and app use			
			Current use (n=1455), n (%)	Past use (n=695), n (%)	*P* value	
**Type of IoT device^a^**						
	PCs	306 (14.2)	194 (13.3)	112 (16.1)	.10	
	Tablet device	184 (8.6)	128 (8.8)	56 (8.1)	.62	
	Smartphone	1805 (84)	1270 (87.3)	535 (77)	<.001	
	Smart ring	91 (4.2)	56 (3.8)	35 (5)	.24	
	Smartwatch	383 (17.8)	287 (19.7)	96 (13.8)	.001	
	Wristband	76 (3.5)	53 (3.6)	23 (3.3)	.79	
	Smart glasses	36 (1.7)	21 (1.4)	15 (2.2)	.30	
	Smart wear	24 (1.1)	15 (1)	9 (1.3)	.75	
	Others	4 (0.2)	3 (0.2)	1 (0.1)	≥.99	
**Frequency of use per week**						<.001
	Daily	399 (18.6)	219 (15.1)	180 (25.9)		
	2-3 days	507 (23.6)	296 (20.3)	211 (30.4)		
	4-5 days	368 (17.1)	224 (15.4)	144 (20.7)		
	6-7 days	876 (40.7)	716 (49.2)	160 (23)		
**Duration of use per day (minutes)**						<.001
	<10	1153 (55.1)	802 (55.1)	351 (50.5)		
	10-30	523 (24.3)	319 (21.9)	204 (29.4)		
	30-60	223 (10.4)	139 (9.6)	84 (12.1)		
	>60	251 (11.7)	195 (13.4)	56 (8.1)		
**Duration of use of app or IoT (months)**						<.001
	<1	311 (14.5)	154 (10.6)	157 (22.6)		
	1-3	379 (17.6)	195 (13.4)	184 (26.5)		
	3-6	359 (16.7)	205 (14.1)	154 (22.2)		
	6-12	270 (12.6)	184 (12.6)	86 (12.4)		
	>12	831 (38.7)	717 (49.3)	114 (16.4)		

^a^More than one response allowed.

Table 3 shows the perceived women-specific health problems or symptoms. Of the 10,000 participants, 63.5% (6350/10,000) perceived they had women-specific health problems or symptoms, of which headaches or migraines (2461/10,000, 24.6%) was the most common, followed by menstrual symptoms or disorders (2130/10,000, 21.3%). Compared with past users and never users, current IoT or app users experienced significantly higher numbers of women-specific health problems, with the exception of insomnia and anemia.

**Table 3:** Perceived women-specific health problems or symptoms by Internet of Things (IoT) or app use among Japanese working women.

Perceived women-specific health problems or symptoms	Total sample (n=10,000), n (%)	Experience with health-related IoT or app use			
		Current use (n=1455), n (%)	Past use (n=695), n (%)	Never used (n=7850), n (%)	*P* value
None	3655 (36.5)	410 (28.2)	230 (33.1)	3015 (38.4)	<.001
Menstrual symptoms or disorders	2130 (21.3)	401 (27.6)	177 (25.5)	1552 (19.8)	<.001
Premenstrual syndrome	1832 (18.3)	356 (24.5)	135 (19.4)	1341 (17.1)	<.001
Infertility	174 (1.7)	38 (2.6)	12 (1.7)	124 (1.6)	.02
Pregnancy or childbirth-related symptoms or illnesses	277 (2.8)	67 (4.6)	25 (3.6)	185 (2.4)	<.001
Menopausal symptoms	1045 (10.4)	189 (13)	82 (11.8)	774 (9.9)	.001
Postmenopausal hormone-related issues	284 (2.8)	55 (3.8)	16 (2.3)	213 (2.7)	.054
Mental disorder	1533 (15.3)	283 (19.5)	102 (14.7)	1148 (14.6)	<.001
Insomnia	1192 (11.9)	199 (13.7)	88 (12.7)	905 (11.5)	.055
Headaches or migraines	2461 (24.6)	392 (26.9)	141 (20.3)	1928 (24.6)	.004
Blood flow disorders	1039 (10.4)	187 (12.9)	73 (10.5)	779 (9.9)	.003
Anemia	1103 (11)	184 (12.6)	74 (10.6)	845 (10.8)	.10
Gastrointestinal disorders	1848 (18.5)	304 (20.9)	125 (18)	1419 (18.1)	.04
Nutritional disorders^a^	1342 (13.4)	249 (17.1)	112 (16.1)	981 (12.5)	<.001
Female-specific cancer^b^	201 (2)	56 (3.8)	23 (3.3)	122 (1.6)	<.001
Endometriosis and benign female-specific tumors	498 (5)	97 (6.7)	35 (5)	366 (4.7)	.005
Thyroid disorders	268 (2.7)	54 (3.7)	21 (3)	193 (2.5)	.02
Pelvic floor symptoms or diseases	492 (4.9)	93 (6.4)	49 (7.1)	350 (4.5)	<.001
Others	102 (1)	13 (0.9)	7 (1)	82 (1)	.87

^a^Underweight, obesity, swelling, diet, or nutritional disorders.

^b^Cervical, uterine, ovarian, or breast cancer.

Regarding the purpose of using IoT or apps for health promotion or prevention, current users were more likely to use IoT or apps to prevent nutritional disorders (460/1455, 31.6%; Table 4). Similarly, for the purpose of using IoT or apps to improve symptoms or illnesses, IoT and apps were more often used to improve nutritional disorders (405/1455, 27.8%). The trend observed among past users is noteworthy, with a significant proportion (160/695, 23%) using IoT or apps for health promotion or prevention of nutritional disorders, along with a similar percentage (158/695, 22.7%) using them to improve nutritional disorders.

**Table 4:** Purpose of Internet of Things (IoT) or app use for health promotion or prevention or to improve symptoms or illnesses for Japanese working women.

Purpose of IoT or app use		Total sample (n=10,000), n (%)	Experience with health-related IoT or app use			
			Current use (n=1455), n (%)	Past use (n=695), n (%)		*P* value
**Health promotion or prevention**						
	Menstrual symptoms or disorders	534 (5.3)	375 (25.8)		159 (22.9)	.16
	Premenstrual syndrome	302 (3)	211 (14.5)		91 (13.1)	.42
	Infertility	80 (0.8)	49 (3.4)		31 (4.5)	.26
	Pregnancy or childbirth-related symptoms or illnesses	159 (1.6)	95 (6.5)		64 (9.2)	.03
	Menopausal symptoms	180 (1.8)	122 (8.4)		58 (8.3)	≥.99
	Postmenopausal hormone-related issues	85 (0.9)	59 (4.1)		26 (3.7)	.82
	Mental disorder	223 (2.2)	153 (10.5)		70 (10.1)	.81
	Insomnia	285 (2.9)	203 (14)		82 (11.8)	.19
	Headaches or migraines	213 (2.1)	142 (9.8)		71 (10.2)	.80
	Blood flow disorders	144 (1.4)	103 (7.1)		41 (5.9)	.35
	Anemia	110 (1.1)	68 (4.7)		42 (6)	.21
	Gastrointestinal disorders	211 (2.1)	146 (10)		65 (9.4)	.68
	Nutritional disorders^a^	620 (6.2)	460 (31.6)		160 (23)	<.001
	Female-specific cancer^b^	148 (1.5)	89 (6.1)		59 (8.5)	.052
	Endometriosis and benign female-specific tumors	62 (0.6)	44 (3)		18 (2.6)	.67
	Thyroid disorders	38 (0.4)	27 (1.9)		11 (1.6)	.78
	Pelvic floor symptoms or diseases	54 (0.5)	32 (2.2)		22 (3.2)	.23
	Others	145 (1.5)	122 (8.4)		23 (3.3)	<.001
**Improve symptoms or illnesses**						
	Menstrual symptoms or disorders	347 (3.5)	249 (17.1)		98 (14.1)	.09
	Premenstrual syndrome	239 (2.4)	173 (11.9)		66 (9.5)	.12
	Infertility	59 (0.6)	37 (2.5)		22 (3.2)	.49
	Pregnancy or childbirth-related symptoms or illnesses	71 (0.7)	42 (2.9)		29 (4.2)	.15
	Menopausal symptoms	140 (1.4)	98 (6.7)		42 (6)	.61
	Postmenopausal hormone-related issues	90 (0.9)	61 (4.2)		29 (4.2)	≥.99
	Mental disorder	202 (2)	142 (9.8)		60 (8.6)	.45
	Insomnia	247 (2.5)	161 (11.1)		86 (12.4)	.41
	Headaches or migraines	236 (2.4)	164 (11.3)		72 (10.4)	.58
	Blood flow disorders	168 (1.7)	121 (8.3)		47 (6.8)	.24
	Anemia	130 (1.3)	79 (5.4)		51 (7.3)	.10
	Gastrointestinal disorders	201 (2)	135 (9.3)		66 (9.5)	.93
	Nutritional disorders^a^	563 (5.6)	405 (27.8)		158 (22.7)	.01
	Female-specific cancer^b^	53 (0.5)	36 (2.5)		17 (2.4)	≥.99
	Endometriosis and benign female-specific tumors	63 (0.6)	41 (2.8)		22 (3.2)	.76
	Thyroid disorders	51 (0.5)	31 (2.1)		20 (2.9)	.36
	Pelvic floor symptoms or diseases	77 (0.8)	43 (3)		34 (4.9)	.03
	Others	83 (0.8)	69 (4.7)		14 (2)	.003

^a^Underweight, obesity, swelling, diet or nutritional disorders.

^b^Cervical, uterine, ovarian, or breast cancer.

The 3 most common reasons for discontinuing IoT or app use were “Became too much of a hassle” (255/695, 36.7%), “Perceived ineffectiveness” (130/695, 18.7%), and “Difficulty using the IoT/app” (96/695, 13.8%). Other reasons included “Intended to try it for a trial period and quit” (85/695, 12.2%), “No longer have time” (79/695, 11.4%), “Boredom” (67/695, 9.6%), “Found a better service elsewhere” (64/695, 9.2%), “High price” (56/695, 8.1%), “Dissatisfied with support” (53/695, 7.6%), “Decreased frequency of use” (52/695, 7.5%), “Improved symptoms” (45/695, 6.5%), and “Other” (30/695, 4.3%).

**Figure 1:**
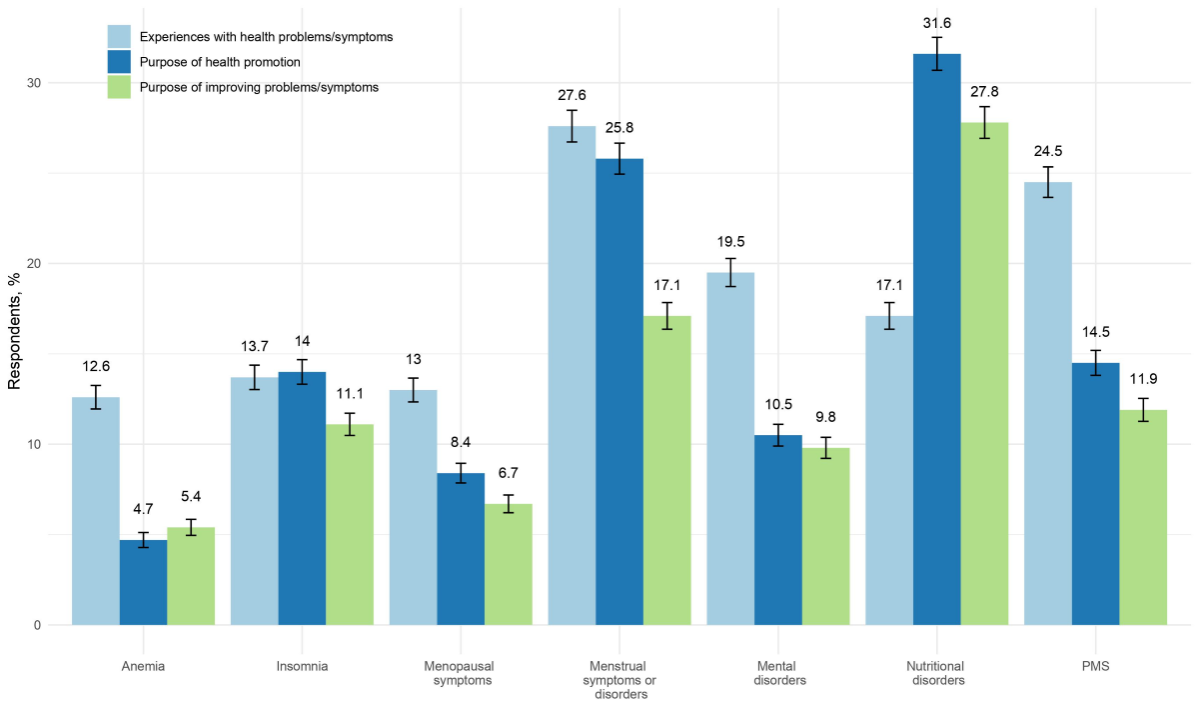
Reasons for using health-related Internet of Things or apps for current users, by perceived health problem among Japanese working women (n=1455), with bars indicating the 95% CIs. PMS: premenstrual syndrome.

Figure 1 compares current IoT and app users by perceived health problems and reasons for the use of IoT or apps in Japan. Among current users, 31.6% (460/1455) of participants stated that they use IoT or apps for health promotion such as nutritional disorders, while only 17.1% (249/1455) of participants actually perceived such problems. Furthermore, although 27.6% (401/1455) of participants were currently experiencing menstrual symptoms or disorders such as irregular menstruation or menstrual pain, only 17.1% (249/1455) of participants used IoT or apps to manage these symptoms. Similarly, 24.5% (356/1455) of participants reported experiencing premenstrual syndrome (PMS), but only 11.9% (173/1455) of participants used IoT or apps to alleviate these symptoms.

**Figure 2:**
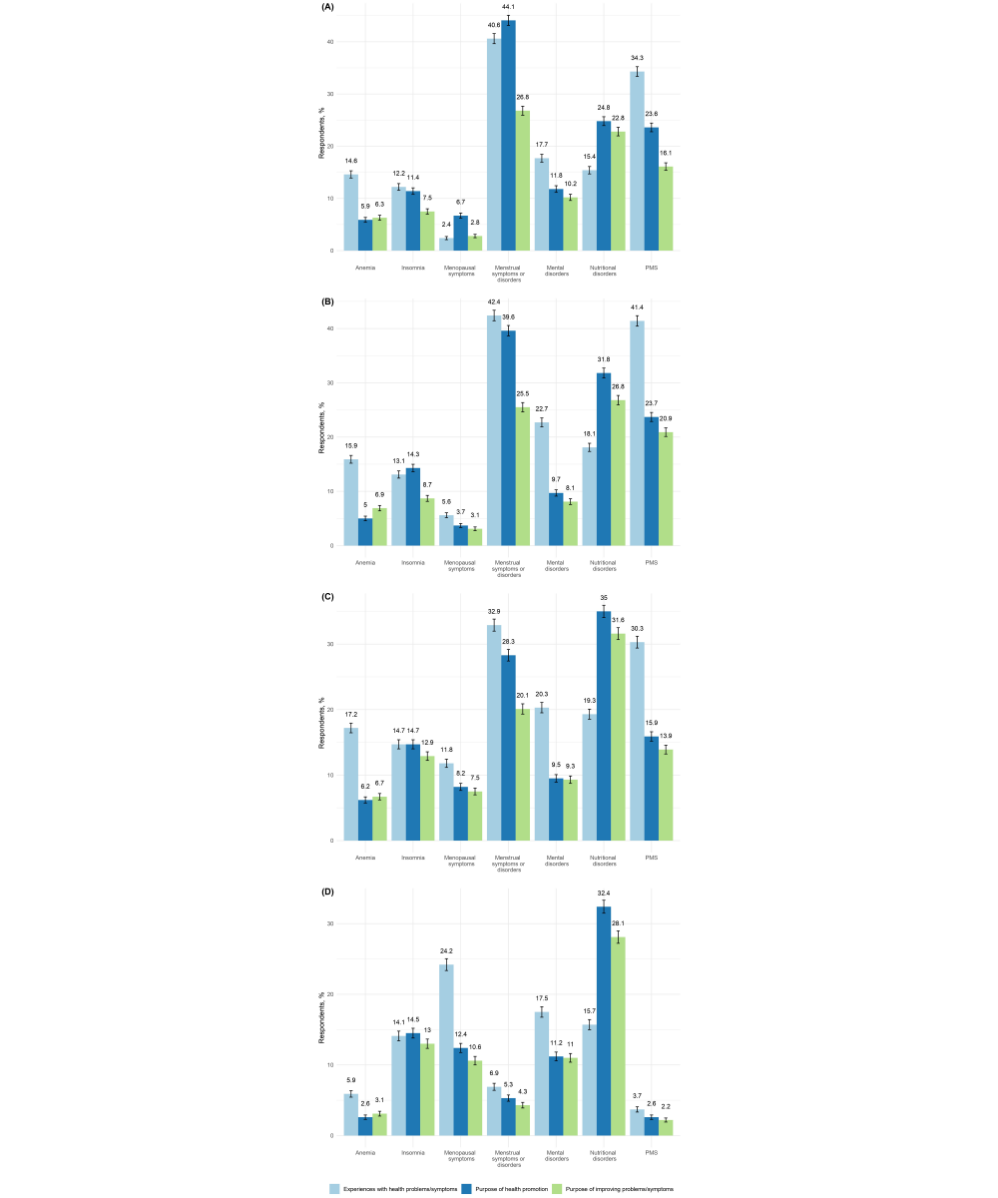
Comparison of participant experiences with domain-specific health problems or symptoms, with the purpose for using health-related Internet of Things or apps by age: (A) 20 years to 29 years (n=254), (B) 30 years to 39 years (n=321), (C) 40 years to 49 years (n=389), (D) 50 years to 69 years (n=491). PMS: premenstrual syndrome. A higher-resolution version of this figure can be found in Multimedia Appendix 2.

Figure 2 compares current users of health-related IoT or apps by perceived health problems and the reason for using IoT or apps, categorized by age group. Menstrual symptoms or disorders and PMS symptoms were more prevalent among individuals aged in their 20s to 40s. In all age groups, using IoT or apps for improvement was more prevalent than using IoT or apps for perceived health problems related to nutritional disorders; in contrast, fewer women aged in their 20s to 40s used them for improvement than for perceived health problems related to menstrual symptoms or disorders and PMS.

### Decision Trees Showing Use of IoT and Apps

**Figure 3:**
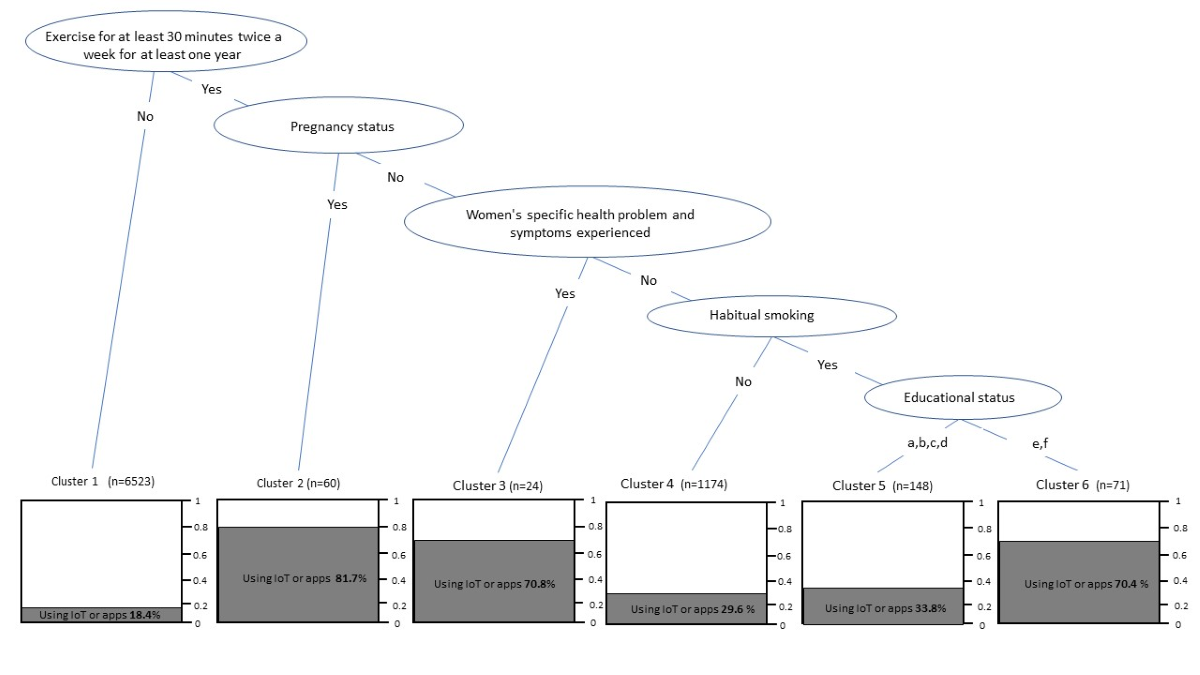
Decision tree analysis using the C5.0 algorithm showing use of Internet of Things (IoT) or apps in 6 clusters of Japanese working women. Educational status: a: junior high school graduate; b: high school graduate; c: associate’s degree; d: some college (no degree); e: undergraduate degree; f: graduate degree.

Figure 3 presents the results of the decision tree analysis using the C5.0 algorithm. We identified 6 clusters with diverse backgrounds showing the use or nonuse of IoT or apps based on the size of clusters with visual representations. The largest cluster, Cluster 1, consisted of 6523 participants, the majority of whom (5323/6523, 81.6%) were non-IoT users who did not exercise for at least 30 minutes twice a week.

The small Cluster 2, comprising 60 participants, consisted of pregnant women who exercised at least twice a week for at least 30 minutes, of whom 82% (40/60) used IoT or apps. The small Cluster 3, with 24 participants, consisted of nonpregnant women who exercised at least twice a week for at least 30 minutes and had women-specific health problems, of whom 71% (17/24) used IoT or apps.

## Discussion

### Summary of Main Findings

Our main findings revealed that 14.6% of participants currently used IoT or apps to manage or control their health problems, while 7% previously used them but had discontinued use (combined 21.6%). When specifically examining the use of IoT or apps by working women in Japan, we noticed low use of IoT or apps to prevent or improve health problems, even among women facing such problems. In other words, a disparity was found between the participants’ experiences with women-specific health problems and the purpose for using IoT or apps. These disparities were particularly evident in relation to symptoms and illnesses related to menstruation and PMS, with all age groups except those aged 50 years to 64 years perceiving them as health concerns. The impact of mobile health on overall health has been extensively studied worldwide [31,32].

A number of studies have investigated the association between women's health and the use of IoT or apps to manage well-being. Many of these studies primarily focused on examining the effectiveness of apps for controlling diseases like gestational diabetes [33] and managing obesity during pregnancy and the perinatal period [34,35]. In contrast, several studies have demonstrated that IoT and apps foster healthy behaviors and exhibit potential for positive health outcomes [36-38]. However, research into the use of IoT and apps among “working” women and its implications for their health remains limited.

In Japan, the ownership of mobile devices has steadily increased, from 94.5% in 2011 to 97.5% in 2022, and the rate of personal internet use reached 84.9% in 2022. Furthermore, 90.1% of individuals use the internet on their smartphone [39].

Regarding trends among IoT and app users, our study revealed that IoT and app users were more likely to have higher levels of education and income than nonusers. This finding aligns with that of a previous study conducted in the United States, which found that individuals with higher education and income were more likely to use health apps [40]. In addition, the decision tree results showed that pregnant women with an exercise habit were more likely to use IoT and apps. Given previous findings that pregnant women were more likely to seek health information [41], it is likely that mothers use IoT or apps to ensure a healthy lifestyle for the birth of their child. On the other hand, the 81.6% of the 10,000 participants in this study who did not have a regular exercise routine did not use IoT or apps for their health problems. An Australian study reported that, among 264 women with menstruation-related conditions, 72% used apps to manage their menstruation, and the majority (91%) expressed a willingness to use apps for menstrual control [42]. Although our study did not inquire about participants’ willingness to use menstruation-related apps, the use of digital health to solve women’s health problems has not noticeably advanced in Japan.

The findings of this study indicate 2 potential reasons for the limited progress in Japan regarding the use of mobile technology using IoT and apps to address women’s health issues. First, it is possible that suitable IoT and apps designed specifically for Japanese women have not yet been developed or are not widely known. IoT and apps for managing menstrual pain and other discomfort should consider language and cultural variations, but the worldwide availability of evidence-based pain and symptom management is still insufficient [43]. Furthermore, there is a lack of clinically informed self-management IoT and apps for effective pain control developed in collaboration with medical professionals [44]. Second, even if products supporting women’s health are developed, current regulations are not conducive to their sale in Japan. Currently, specialized products that provide interactive digital health applications for women’s health are gaining attention. These products encompass apps or high-tech services tailored specifically to women’s health needs, such as digital fertility or menstruation tracking, as well as solutions for pregnancy, postpartum, and menopause. The term “FemTech” was coined by Danish-born Ida Ting, the founder of Clue, a period and ovulation tracking app established in Germany in 2013 [45]. However, FemTech products are still in the early stages of development in Japan and face accessibility challenges [46]. One reason is that the devices, apps, or programs that combine these elements are categorized as “medical devices” and are subject to regulation under the “Act on Quality, Efficacy, and Safety Assurance of Drugs and Medical Devices” (Pharmaceutical Affairs Law) [47]. Unless a FemTech product or service has received regulatory approval, the product—no matter whether it is used to improve health and not treat diseases—cannot report its efficacy and safety on the package, even if it has been scientifically proven safe and effective. Such factors may partially contribute to the apparent discrepancy between the symptoms experienced by working women and the use of IoT and apps. Introducing a more conducive environment for the development and promotion of FemTech products warrants greater attention from policymakers.

Although the use of IoT or apps is anticipated to enhance women’s health, issues pertaining to data privacy have emerged. Global enforcement of privacy standards remains incomplete. For instance, disclosure of information regarding a woman’s pregnancy to her company’s human resources department poses a potential risk, potentially leading to missed promotion opportunities. Establishing a foundational framework for safeguarding this exceptionally sensitive health information and user rights is a pivotal challenge for the future.

### Strengths and Limitations of the Study

This study represents the first large-scale internet survey, encompassing 10,000 cases, on the current use of health-related IoT and apps for health management among working women in Japan. The findings hold significant utility for researchers, policymakers, and other stakeholders interested in this domain. However, this study has several limitations that should be acknowledged. First, the study used a cross-sectional design, which means that it cannot establish causality between IoT or app use and the health status of participants. Furthermore, the study specifically targeted Japanese women, and the generalizability of the findings to other populations needs to be further investigated. Second, there are potential biases inherent in the study. For instance, respondents who had previously used (but stopped using) IoT or apps were asked about the purpose and frequency of their past use, introducing the possibility of recall bias. Additionally, the question regarding exercise duration, “exercise for at least 30 minutes twice a week for at least one year,” lacks a mechanism for verifying if participants have truly exercised for 1 year.

However, it should be noted that, since IoT devices typically record log histories, self-referencing is relatively straightforward, minimizing the impact of recall bias. Additionally, the nature of an internet survey introduces limitations. There is selection bias in that busy women may not have had the time to respond to the survey and those without internet access were excluded. To address this limitation, we are currently planning a new survey using mail-based methods.

### Conclusion

In conclusion, this study examined the use of IoT and apps to manage the health of working women in Japan. The findings showed that a smaller proportion of working women reported using IoT or apps for health care purposes. There was a notable discrepancy between the participants’ perceived health concerns and their actual use of IoT and apps, particularly regarding symptoms related to menstruation and PMS. The study also highlighted that women in their 30s to 40s were more likely to use IoT and apps to address health issues. Overall, our study provides insights into the current state of IoT or app use among working women and the need for further research.

## Appendix

Multimedia Appendix 1 Survey on the Use of Applications and IoT by Working Women.

Multimedia Appendix 2 Comparison of participant experience with domain-specific health problems or symptoms with the purpose for using health-related Internet of Things or apps by age: (A) 20 years to 29 years (n=254), (B) 30 years to 39 years (n=321), (C) 40 years to 49 years (n=389), (D) 50 years to 69 years (n=491). PMS: premenstrual syndrome.

## Abbreviations

CHERRIES: Checklist for Reporting Results of Internet E-Surveys
IoT: Internet of Things
PMS: premenstrual syndrome

## Data Availability

The data sets generated and analyzed during this study are available from the corresponding author upon reasonable request.
