# Correction: Current Use and Discrepancies in the Adoption of Health-Related Internet of Things and Apps Among Working Women in Japan: Large-Scale, Internet-Based, Cross-Sectional Survey

**DOI:** 10.2196/65194

**Published:** 2024-08-21

**Authors:** Kiriko Sasayama, Etsuko Nishimura, Noyuri Yamaji, Erika Ota, Hisateru Tachimori, Ataru Igarashi, Naoko Arata, Daisuke Yoneoka, Eiko Saito

**Affiliations:** 1 Sustainable Society Design Center Graduate School of Frontier Sciences The University of Tokyo Kashiwa Japan; 2 Faculty of Nursing Komazawa Women’s University Tokyo Japan; 3 Graduate School of Nursing Science St. Luke's International University Tokyo Japan; 4 Institute of Clinical Epidemiology Showa University Tokyo Japan; 5 Graduate School of Medicine The University of Tokyo Tokyo Japan; 6 Institute for Global Health Policy Research, Bureau of International Health Cooperation National Center for Global Health and Medicine Tokyo Japan; 7 Department of Health Policy and Management Keio University School of Medicine Tokyo Japan; 8 Public Health, School of Medicine Medical Course Yokohama City University Yokohama Japan; 9 Center for Maternal-Fetal-Neonatal and Reproductive Medicine National Center for Child Health and Development Tokyo Japan; 10 Center for Surveillance, Immunization, and Epidemiologic Research National Institute of Infectious Diseases Tokyo Japan

In “Current Use and Discrepancies in the Adoption of Health-Related Internet of Things and Apps Among Working Women in Japan: Large-Scale, Internet-Based, Cross-Sectional Survey” (JMIR Public Health Surveill 2024;10:e51537) the authors noted one error.

In the originally published paper the first authors name appeared as “*Kirio Sasayama*”. This has been corrected to “*Kiriko Sasayama*”.

The correction will appear in the online version of the paper on the JMIR Publications website on August 21^st^ 2024, together with the publication of this correction notice. Because this was made after submission to PubMed, PubMed Central, and other full-text repositories, the corrected article has also been resubmitted to those repositories.

