# Supplementary material for: Current Use and Discrepancies in the Adoption of Health-Related Internet of Things and Apps Among Working Women in Japan: Large-Scale, Internet-Based, Cross-Sectional Survey
Source: JMIR Public Health Surveill. 2024 Jul 31;10:e51537. doi: 10.2196/51537 (PMC11325101; doi:10.2196/51537)
Supplement: Multimedia Appendix 1 [file publichealth_v10i1e51537_app1.docx]

**Survey on the Use of Applications and IoT by Working Women**

**Screening Questions**

1. Please tell us your gender.
   - Male
   - Female
   - Comment: Exclude males
2. Please tell us your age.
   - 19 or younger
   - 20-29
   - 30-39
   - 40-49
   - 50-64
   - 65 or older
   - Comment: Exclude those 19 or younger and 65 or older
3. Please tell us your place of residence (prefecture).
   - 47 prefectures
   - Categorized by region (Hokkaido, Tohoku, Hokuriku-Koshinetsu, Kanto, Chubu, Kinki, Chugoku-Shikoku, Kyushu-Okinawa)
4. Are you currently employed?
   - Business owner/Executive
   - Full-time employee
   - Contract employee
   - Temporary employee
   - Public servant (excluding teachers)
   - Teacher
   - Healthcare professional
   - Professional (CPA, lawyer, tax accountant, judicial scrivener)
   - Self-employed
   - Part-time/Arbeit
   - Housewife
   - Student
   - Unemployed
   - Comment: Exclude housewives, students, and unemployed

**Section 1: Basic Attributes and Health Status**

Please answer the following questions about yourself and your health.

1. Do you currently live with a spouse? (Including those living as a couple without submitting a marriage certificate)
   - Yes
   - No
2. Do you have any children living with you?
   - No
   - 1
   - 2
   - 3
   - 4
   - 5 or more
3. If you have children, please tell us the age of each child.
4. Is there anyone living with you who needs care?
   - No
   - 1
   - 2 or more
5. If you have someone who needs care, please specify who. (Multiple answers allowed)
   - Child
   - Spouse
   - Parents (including in-laws)
   - Grandparents (including in-laws)
   - Siblings
   - Others FA()
6. Do you have anyone close to you who helps with childcare or caregiving? (Multiple answers allowed)
   - No
   - Public support (home visit services, daycare services, etc.)
   - Private support
   - Spouse
   - Nearby relatives
   - Others FA()
7. Please tell us your household income (living together, sharing household expenses).
   - Less than 2 million yen
   - 2-4 million yen
   - 4-6 million yen
   - 6-8 million yen
   - 8-10 million yen
   - 10-12 million yen
   - 12-14 million yen
   - More than 14 million yen
8. What is your highest level of education?
   - Junior high school
   - High school
   - Junior college
   - Vocational school
   - University
   - Graduate school (Master's/Doctorate)
9. Are you currently pregnant?
   - Yes
   - No
10. Do you have any diagnosed or ongoing conditions? (Multiple answers allowed)
    - None
    - Infertility (including unexplained infertility)
    - Female cancers or cancers common in women (cervical cancer, uterine cancer, ovarian cancer, breast cancer)
    - Hypertension
    - Diabetes
    - Hyperlipidemia
    - Stroke (cerebral hemorrhage, cerebral infarction, etc.)
    - Heart disease (angina, myocardial infarction, etc.)
    - Chronic kidney disease, renal failure
    - Others FA()
11. What is your current work schedule?
    - Day shift only
    - Night shift only
    - Shift work (both day and night shifts)
12. What is your average daily working hours over the past month at your current workplace?
    - Less than 6 hours
    - 6-8 hours
    - 8-10 hours
    - More than 10 hours
13. What is your average number of working days per week over the past month at your current workplace?
    - Less than 3 days
    - 3-5 days
    - 5 days
    - More than 6 days
14. Do you currently smoke regularly? (Defined as smoking more than 100 cigarettes in total or for more than 6 months, and still smoking in the past month)
    - Yes
    - No
15. Have you gained more than 10 kg from your weight at age 20?
    - Yes
    - No
16. Do you engage in light exercise that makes you sweat for 30 minutes or more at least twice a week for over a year?
    - Yes
    - No
17. Do you engage in physical activities such as walking for an hour or more daily in your everyday life?
    - Yes
    - No
18. Do you walk faster compared to people of the same age and gender?
    - Yes
    - No
19. How would you describe your chewing ability when eating?
    - Can chew anything
    - Have concerns about teeth/gums/bite, sometimes difficult to chew
    - Can hardly chew
20. Do you eat faster compared to others?
    - Fast
    - Normal
    - Slow
21. Do you eat dinner within 2 hours before bedtime at least 3 times a week?
    - Yes
    - No
22. Do you consume snacks or sweet drinks besides the three main meals?
    - Every day
    - Sometimes
    - Hardly ever
23. Do you skip breakfast at least 3 times a week?
    - Yes
    - No
24. How often do you drink alcohol (sake, shochu, beer, spirits, etc.)?
    - Every day
    - Sometimes
    - Hardly ever (cannot drink)
25. For those who drink alcohol, how much do you drink per day on drinking days? Standard measure: 1 go (180 ml) of sake equals a medium bottle of beer (about 500 ml), 25-degree shochu (110 ml), a double whiskey (60 ml), or two glasses of wine (240 ml).
    - Less than 1 go
    - 1-2 go
    - 2-3 go
    - More than 3 go
26. Do you get enough rest from sleep?
    - Yes
    - No
27. What is your average sleep time per week over the past month?
    - Less than 5 hours
    - 5-6 hours
    - 6-7 hours
    - 7-8 hours
    - More than 8 hours
28. Please select the specific health issues or symptoms that you are currently experiencing, specific to women or commonly seen in women. (Multiple answers allowed)
    - None
    - Menstrual-related symptoms or diseases (irregular menstruation, menstrual pain)
    - PMS (premenstrual syndrome)
    - Female cancers or cancers common in women (cervical cancer, uterine cancer, ovarian cancer, breast cancer)
    - Pregnancy or childbirth-related symptoms or diseases (morning sickness, miscarriage, preterm birth, stillbirth, postpartum depression, etc.)
    - Menopausal disorders (hot flashes, sweating, irregular menstruation, dizziness, numbness in hands and feet, etc.)
    - Mental disorders (anxiety, restlessness, depression, etc.)
    - Insomnia
    - Infertility
    - Endometriosis or benign tumors common in women (ovarian cyst, uterine fibroid, etc.)
    - Circulatory disorders such as chills or hot flashes
    - Anemia
    - Gastrointestinal disorders such as constipation or diarrhea
    - Headache, migraine
    - Symptoms or diseases caused by decreased female hormones after menopause (osteoporosis, joint pain, atherosclerosis, diabetes, increased risk of dementia, etc.)
    - Thinness, obesity, swelling, diet or nutritional disorders
    - Thyroid disease (Graves' disease, Hashimoto's disease, etc.) or autoimmune diseases
    - Pelvic floor disorders (frequent urination, urinary incontinence, pelvic organ prolapse, etc.)
    - Others FA()
29. Please select the specific health issues or symptoms that you have experienced at your workplace, specific to women or commonly seen in women. (Multiple answers allowed)
    - None
    - Menstrual-related symptoms or diseases (irregular menstruation, menstrual pain)
    - PMS (premenstrual syndrome)
    - Female cancers or cancers common in women (cervical cancer, uterine cancer, ovarian cancer, breast cancer)
    - Pregnancy or childbirth-related symptoms or diseases (morning sickness, miscarriage, preterm birth, stillbirth, postpartum depression, etc.)
    - Menopausal disorders (hot flashes, sweating, irregular menstruation, dizziness, numbness in hands and feet, etc.)
    - Mental disorders (anxiety, restlessness, depression, etc.)
    - Insomnia
    - Infertility
    - Endometriosis or benign tumors common in women (ovarian cyst, uterine fibroid, etc.)
    - Circulatory disorders such as chills or hot flashes
    - Anemia
    - Gastrointestinal disorders such as constipation or diarrhea
    - Headache, migraine
    - Symptoms or diseases caused by decreased female hormones after menopause (osteoporosis, joint pain, atherosclerosis, diabetes, increased risk of dementia, etc.)
    - Thinness, obesity, swelling, diet or nutritional disorders
    - Thyroid disease (Graves' disease, Hashimoto's disease, etc.) or autoimmune diseases
    - Pelvic floor disorders (frequent urination, urinary incontinence, pelvic organ prolapse, etc.)
    - Others FA()
30. How satisfied are you with your current life overall? Rate from 0 (not satisfied at all) to 10 (very satisfied).
31. How valuable do you find the activities you do in your current life overall? Rate from 0 (not valuable at all) to 10 (very valuable).
32. How happy did you feel yesterday? Rate from 0 (not happy at all) to 10 (very happy).
33. How anxious did you feel yesterday? Rate from 0 (not anxious at all) to 10 (very anxious).
34. How satisfied are you with your current workplace overall? Rate from 0 (not satisfied at all) to 10 (very satisfied).
35. How satisfied are you with your leisure time overall? Rate from 0 (not satisfied at all) to 10 (very satisfied).
36. Please specify the activities you are engaged in outside of work. (Multiple answers allowed)
    - None
    - Volunteering
    - Sports
    - Arts and cultural activities
    - Others FA()
37. Where do you get health-related information from? (Multiple answers allowed)
    - Do not obtain information
    - Healthcare professionals
    - Health fairs and events
    - Books (newspapers, magazines, scientific papers, etc.)
    - TV and radio
    - Online news
    - Internet search engines (Google, Yahoo, etc.)
    - SNS
    - Medical information websites (Medical Note, MEDLEY, Apitaru, etc.)
    - Blogs
    - Government and municipal information
    - Family
    - Friends
    - Others FA()
38. How much do you trust information from the following sources? Choose from four levels.
    - Healthcare professionals
      1. Do not trust at all
      2. Somewhat trust
      3. Trust
      4. Trust very much
    - Health fairs and events
      1. Do not trust at all
      2. Somewhat trust
      3. Trust
      4. Trust very much
    - Books (newspapers, magazines, scientific papers, etc.)
      1. Do not trust at all
      2. Somewhat trust
      3. Trust
      4. Trust very much
    - TV and radio
      1. Do not trust at all
      2. Somewhat trust
      3. Trust
      4. Trust very much
    - Online news
      1. Do not trust at all
      2. Somewhat trust
      3. Trust
      4. Trust very much
    - Internet search engines (Google, Yahoo, etc.)
      1. Do not trust at all
      2. Somewhat trust
      3. Trust
      4. Trust very much
    - SNS
      1. Do not trust at all
      2. Somewhat trust
      3. Trust
      4. Trust very much
    - Medical information websites (Medical Note, MEDLEY, Apitaru, etc.)
      1. Do not trust at all
      2. Somewhat trust
      3. Trust
      4. Trust very much
    - Blogs
      1. Do not trust at all
      2. Somewhat trust
      3. Trust
      4. Trust very much
    - Government and municipal information
      1. Do not trust at all
      2. Somewhat trust
      3. Trust
      4. Trust very much
    - Family
      1. Do not trust at all
      2. Somewhat trust
      3. Trust
      4. Trust very much
    - Friends
      1. Do not trust at all
      2. Somewhat trust
      3. Trust
      4. Trust very much
39. Are you thinking about improving your lifestyle habits such as exercise and diet?
    - Do not intend to improve
    - Intend to improve (within approximately 6 months)
    - Intend to improve soon (within approximately 1 month) and have started gradually
    - Have already been working on it (less than 6 months)
    - Have already been working on it (more than 6 months)

**Section 2: Usage of Applications and IoT**

Please answer about your usage of health-related applications and Internet of Things (IoT: smartwatches, etc.) devices.

1. Do you use health-related applications or IoT?
   - Currently using
   - Used before but stopped
   - Never used
2. What is the purpose of using (or having used) these applications or IoT devices? (Multiple answers allowed)
   - For health promotion and prevention (Select the symptoms/diseases you are trying to promote/prevent)
     1. Menstrual-related symptoms or diseases (irregular menstruation, menstrual pain)
     2. PMS (premenstrual syndrome)
     3. Female cancers or cancers common in women (cervical cancer, uterine cancer, ovarian cancer, breast cancer)
     4. Pregnancy or childbirth-related symptoms or diseases (morning sickness, miscarriage, preterm birth, stillbirth, postpartum depression, etc.)
     5. Menopausal disorders (hot flashes, sweating, irregular menstruation, dizziness, numbness in hands and feet, etc.)
     6. Mental disorders (anxiety, restlessness, depression, etc.)
     7. Insomnia
     8. Infertility
     9. Endometriosis or benign tumors common in women (ovarian cyst, uterine fibroid, etc.)
     10. Circulatory disorders such as chills or hot flashes
     11. Anemia
     12. Gastrointestinal disorders such as constipation or diarrhea
     13. Headache, migraine
     14. Symptoms or diseases caused by decreased female hormones after menopause (osteoporosis, joint pain, atherosclerosis, diabetes, increased risk of dementia, etc.)
     15. Thinness, obesity, swelling, diet or nutritional disorders
     16. Thyroid disease (Graves' disease, Hashimoto's disease, etc.) or autoimmune diseases
     17. Pelvic floor disorders (frequent urination, urinary incontinence, pelvic organ prolapse, etc.)
     18. Others FA()
   - For symptom improvement (Select the symptoms/diseases you are trying to improve)
     1. Menstrual-related symptoms or diseases (irregular menstruation, menstrual pain)
     2. PMS (premenstrual syndrome)
     3. Female cancers or cancers common in women (cervical cancer, uterine cancer, ovarian cancer, breast cancer)
     4. Pregnancy or childbirth-related symptoms or diseases (morning sickness, miscarriage, preterm birth, stillbirth, postpartum depression, etc.)
     5. Menopausal disorders (hot flashes, sweating, irregular menstruation, dizziness, numbness in hands and feet, etc.)
     6. Mental disorders (anxiety, restlessness, depression, etc.)
     7. Insomnia
     8. Infertility
     9. Endometriosis or benign tumors common in women (ovarian cyst, uterine fibroid, etc.)
     10. Circulatory disorders such as chills or hot flashes
     11. Anemia
     12. Gastrointestinal disorders such as constipation or diarrhea
     13. Headache, migraine
     14. Symptoms or diseases caused by decreased female hormones after menopause (osteoporosis, joint pain, atherosclerosis, diabetes, increased risk of dementia, etc.)
     15. Thinness, obesity, swelling, diet or nutritional disorders
     16. Thyroid disease (Graves' disease, Hashimoto's disease, etc.) or autoimmune diseases
     17. Pelvic floor disorders (frequent urination, urinary incontinence, pelvic organ prolapse, etc.)
     18. Others FA()
3. Please specify the IoT devices you use (or used) to operate health-related applications (Multiple answers allowed)
   - PC
   - Tablet
   - Smartphone
   - Smart ring
   - Smartwatch
   - Wristband
   - Smart glasses
   - Smartwear
   - Others
4. How many days per week do you use (or used) the applications or IoT devices?
   - 1 day
   - 2-3 days
   - 4-5 days
   - 6-7 days
5. How much time do you spend per day using (or used) the applications or IoT devices?
   - Within 10 minutes
   - 11-30 minutes
   - 31-60 minutes
   - More than 61 minutes
6. How long have you been using (or used) the applications or IoT devices?
   - Within 1 month
   - 2-3 months
   - 4-6 months
   - More than 1 year
7. Please specify the applications you use (or used). (Multiple answers allowed)
   - SNS (YouTube, Facebook, Twitter, TikTok, Instagram, LINE, etc.)
   - Asken
   - FiNC
   - Calo Mama
   - Calomill
   - Pause and Class
   - Welby My Carte
   - SmartDiet
   - LunaLuna
   - Clue Period Tracker
   - Others FA()
8. How satisfied are you with the applications you use (or used)? (Multiple answers allowed)
   - SNS (YouTube, Facebook, Twitter, TikTok, Instagram, LINE, etc.)
     1. Very dissatisfied
     2. Dissatisfied
     3. Satisfied
     4. Very satisfied
   - Asken
     1. Very dissatisfied
     2. Dissatisfied
     3. Satisfied
     4. Very satisfied
   - FiNC
     1. Very dissatisfied
     2. Dissatisfied
     3. Satisfied
     4. Very satisfied
   - Calo Mama
     1. Very dissatisfied
     2. Dissatisfied
     3. Satisfied
     4. Very satisfied
   - Calomill
     1. Very dissatisfied
     2. Dissatisfied
     3. Satisfied
     4. Very satisfied
   - Pause and Class
     1. Very dissatisfied
     2. Dissatisfied
     3. Satisfied
     4. Very satisfied
   - Welby My Carte
     1. Very dissatisfied
     2. Dissatisfied
     3. Satisfied
     4. Very satisfied
   - SmartDiet
     1. Very dissatisfied
     2. Dissatisfied
     3. Satisfied
     4. Very satisfied
   - LunaLuna
     1. Very dissatisfied
     2. Dissatisfied
     3. Satisfied
     4. Very satisfied
   - Clue Period Tracker
     1. Very dissatisfied
     2. Dissatisfied
     3. Satisfied
     4. Very satisfied
   - Others FA()
     1. Very dissatisfied
     2. Dissatisfied
     3. Satisfied
     4. Very satisfied
9. For those who answered "Used before but stopped" for question 1, please specify up to 3 reasons why you stopped using the applications or IoT devices. (Multiple answers allowed)
   - Did not feel effective
   - Dissatisfied with support
   - Not user-friendly
   - Found a better service
   - Expensive
   - Planned to stop after trial
   - Symptoms improved
   - Used less frequently
   - No longer had time
   - Became cumbersome
   - Lost interest
   - Others FA()
