# Supplementary figures and images for: Current Use and Discrepancies in the Adoption of Health-Related Internet of Things and Apps Among Working Women in Japan: Large-Scale, Internet-Based, Cross-Sectional Survey
Source: JMIR Public Health Surveill. 2024 Jul 31;10:e51537. doi: 10.2196/51537 (PMC11325101; doi:10.2196/51537)

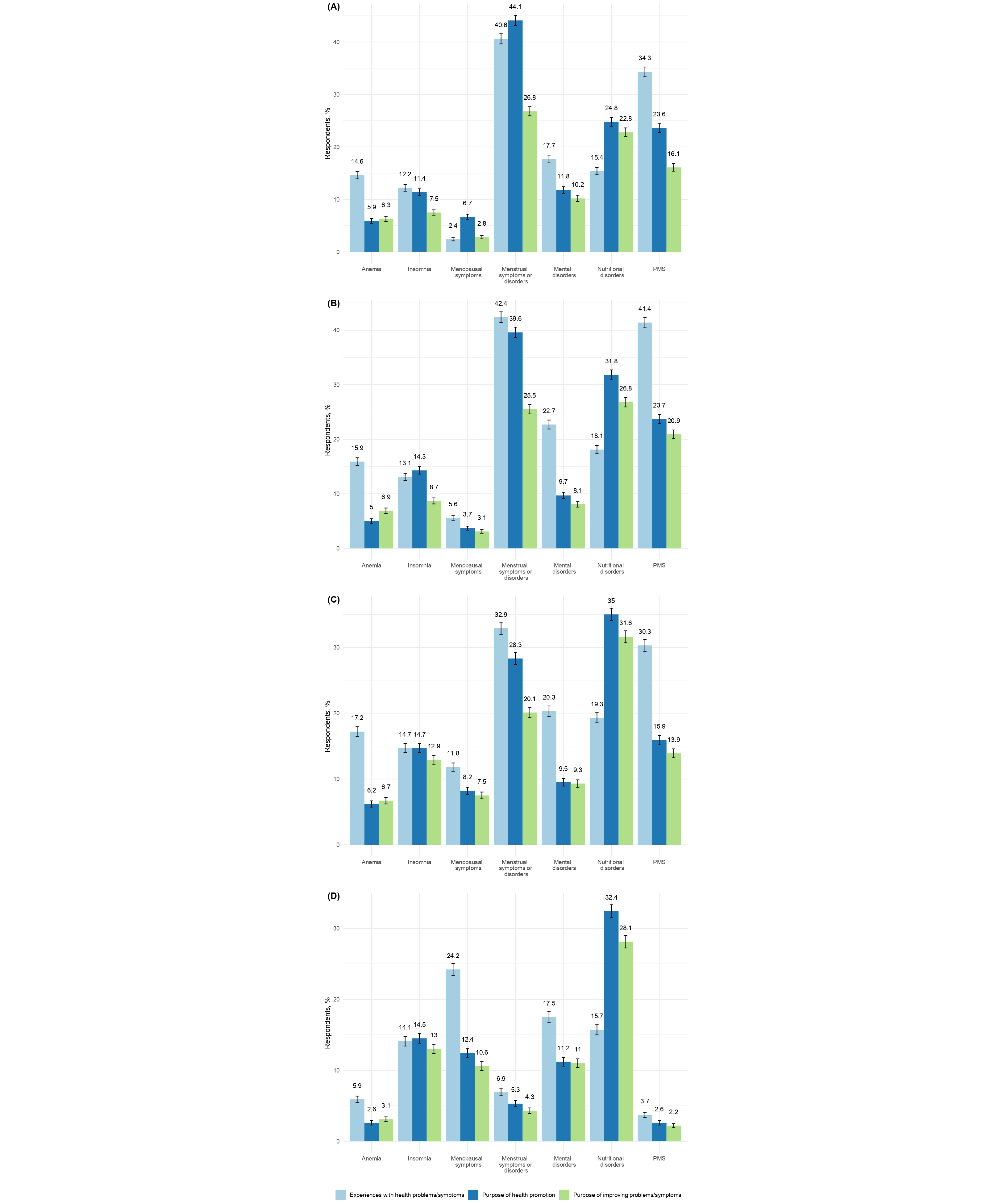

Supplement: Multimedia Appendix 2 [file publichealth_v10i1e51537_app2.png]
